# RELIGIOSITY AND SENSE OF PURPOSE IN WHITE AND BLACK OLDER ADULTS

**DOI:** 10.1093/geroni/igad104.3565

**Published:** 2023-12-21

**Authors:** Selin Toprakkiran, Megan Wilson, Thomas Oltmanns, Ryan Bogdan, Patrick Hill

**Affiliations:** Washington University in St. Louis, St. Louis, Missouri, United States; Washington University in St. Louis, St. Louis, Missouri, United States; Washington University in St. Louis, St. Louis, Missouri, United States; Washington University in St. Louis, St. Louis, Missouri, United States; Washington University in St. Louis, St. Louis, Missouri, United States

## Abstract

Religiosity is a key factor in the lives of many older Americans, and it may help people develop a sense of purpose. This association is highly nuanced, across groups and measures. For instance, religiosity plays a different role across racial/ethnic groups in the United States, which may influence its association with sense of purpose. To examine whether the relationship between religiosity and sense of purpose differs across White and Black Americans, we used the St. Louis Personality and Aging Network data (N = 732), collected from older adults (M age = 67.96; SD age = 4.31; 76.37% White, 23.63% Black American). We used data from multiple waves to examine religiosity, sense of purpose, and social engagement. To disentangle different aspects of religiosity, we included three items focusing on the meaning of religion to one’s life, participation in religious activities, and how much religion influences one’s daily life. Black participants scored higher on all religiosity items compared to White participants. Nevertheless, the relationship between religiosity and sense of purpose did not differ based on one’s racial/ethnic identity or the aspect of religiosity. Additionally, social support did not moderate relationships between religiosity and sense of purpose, suggesting that these relationships are similar regardless of whether one perceives more or less social support from others. These findings add to our understanding of when and for whom religiosity correlates with purpose among older adults, and future directions include examining the influence of religiosity on changes in purpose over time.

